# Tyrosyl-DNA Phosphodiesterase 1 and Topoisomerase I Activities as Predictive Indicators for Glioblastoma Susceptibility to Genotoxic Agents

**DOI:** 10.3390/cancers11101416

**Published:** 2019-09-23

**Authors:** Wenjie Wang, Monica Rodriguez-Silva, Arlet M. Acanda de la Rocha, Aizik L. Wolf, Yanhao Lai, Yuan Liu, William C. Reinhold, Yves Pommier, Jeremy W. Chambers, Yuk-Ching Tse-Dinh

**Affiliations:** 1 Biomolecular Sciences Institute, Florida International University, Miami, FL 33199, USA; wwang036@fiu.edu (W.W.); yalai@fiu.edu (Y.L.); yualiu@fiu.edu (Y.L.); 2 Department of Chemistry and Biochemistry, Florida International University, Miami, FL 33199, USA; 3 Department of Environmental Health Sciences, Florida International University, Miami, FL 33199, USA; mrodr204@fiu.edu (M.R.-S.); aacandad@fiu.edu (A.M.A.d.l.R.); 4 Department of Neurosurgery, Miami Neuroscience Center at Larkin, South Miami, FL 33143, USA; aizikwolf@hotmail.com; 5 Developmental Therapeutic Branch, Center for Cancer Research, National Cancer Institute, National Institutes of Health, Bethesda, MD 20892 USA; wcr@mail.nih.gov (W.C.R.); yves.pommier@nih.gov (Y.P.)

**Keywords:** topoisomerase, TOP1, TDP1, irinotecan, risk-benefit, predictive indicator

## Abstract

Glioblastoma (GBM) patients have an estimated survival of ~15 months with treatment, and the standard of care only modestly enhances patient survival. Identifying biomarkers representing vulnerabilities may allow for the selection of efficacious chemotherapy options to address personalized variations in GBM tumors. Irinotecan targets topoisomerase I (TOP1) by forming a ternary DNA–TOP1 cleavage complex (TOP1cc), inducing apoptosis. Tyrosyl-DNA phosphodiesterase 1 (TDP1) is a crucial repair enzyme that may reduce the effectiveness of irinotecan. We treated GBM cell lines with increasing concentrations of irinotecan and compared the IC_50_ values. We found that the TDP1/TOP1 activity ratio had the strongest correlation (Pearson correlation coefficient R = 0.972, based on the average from three sets of experiments) with IC_50_ values following irinotecan treatment. Increasing the TDP1/TOP1 activity ratio by the ectopic expression of wild-type TDP1 increased in irinotecan IC_50_, while the expression of the TDP1 catalytic-null mutant did not alter the susceptibility to irinotecan. The TDP1/TOP1 activity ratio may be a new predictive indicator for GBM vulnerability to irinotecan, allowing for the selection of individual patients for irinotecan treatment based on risk–benefit. Moreover, TDP1 inhibitors may be a novel combination treatment with irinotecan to improve GBM patient responsiveness to genotoxic chemotherapies.

## 1. Introduction

Glioblastoma (GBM) is the most common and aggressive primary brain tumor in adults, with a dismal prognosis indicated by a 14.6-month median survival after surgical resection, radiotherapy, and chemotherapy treatment with temozolomide (TMZ) [1]. Irinotecan (IRT) is used to treat metastatic colorectal cancer and small cell lung cancer (SCLC) [2,3]. Irinotecan has some capacity to cross the blood–brain barrier (BBB) [4], and was shown to be effective against recurrent malignant glioma in early phase II clinical trials [5,6], although other clinical trials could not confirm the activity from irinotecan monotherapy [7,8]. Studies of irinotecan in combination therapy have yielded modest activity as well [7,9,10,11]. These seemingly discrepant results may indicate an underlying variability in the susceptibility of patients in each trial towards the genotoxic chemotherapy. Thus, to improve clinical outcomes and minimize toxic side effects, identifying biomarkers for GBM susceptibility to irinotecan would be instrumental for selecting patients for irinotecan treatment.

**Figure 1:**
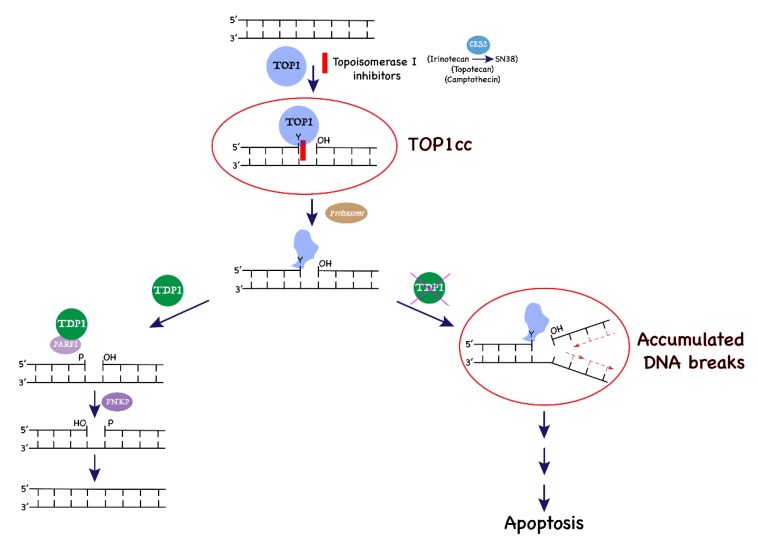
The action of topoisomerase I (TOP1) poison inhibitors and role of tyrosyl-DNA phosphodiesterase 1 (TDP1) in the repair of TOP1 cleavage complexes (TOP1cc) to prevent cell death.

Irinotecan, its active metabolite SN-38, and topotecan are chemotherapeutic camptothecin derivatives (CPTs) acting as topoisomerase I (TOP1) poisons [12] (Figure 1). Human TOP1 is vital for replication and transcription, as it relaxes DNA superhelical tension through phosphodiester bond cleavage, strand rotation, and DNA resealing steps [13]. During the DNA cleavage step, TOP1 covalently attaches to the 3’-end of DNA, with its catalytic tyrosine residue forming reversible DNA–TOP1 cleavage complexes (TOP1cc). Stabilization of the TOP1cc by TOP1 poisons generates deleterious single-strand breaks (SSBs) [14]. A collision between the replication fork and TOP1cc produces DNA double-strand breaks (DSBs) and triggers cell death [15,16]. The lethal TOP1cc can be trapped by endogenous DNA lesions and oxidative stress-induced base modifications [13,17,18,19]. Therefore, the irreversible TOP1cc must be countered by the endogenous repair mechanism to preserve genome integrity [20,21]. Tyrosyl-DNA phosphodiesterase 1 (TDP1) liberates TOP1 peptide from the 3’ end of DNA by hydrolyzing the phosphotyrosyl bond after proteasome-dependent degradation of TOP1cc [22,23] (Figure 1).

Human TDP1, a member of the phospholipase D (PLD) superfamily, has two crucial catalytic residues, H263 and H493 [24]. A homozygous TDP1 H493R mutation is associated with familial spinocerebellar ataxia with axonal neuropathy (SCAN1) [25,26]. Lymphoblastoid cells derived from SCAN1 patients are hypersensitive to CPTs, resulting from a 25-fold reduction in TDP1 activity [27], implying that TDP1 activity may suppress the effectiveness of CPTs. Moreover, TDP1 deficiency in *TDP1* knockdown cells confers enhanced cytotoxic response to CPT, whereas the depletion of TOP1 rescues cells from CPT-induced cell death [28,29]. Additionally, phosphorylation of the TOP1 conserved-core domain (residue 198–651) elevates TOP1 activity, which in turn reduces cellular resistance to CPT [30,31]. The activity levels of both TOP1 and TDP1 may affect the anticancer efficacy of TOP1 poisons.

In this study, we measured the protein concentrations and activity of both TOP1 and TDP1 in nine GBM cell lines. Elevated TOP1 levels and a diminished TDP1 abundance were observed in GBM lines compared to fetal normal human astrocyte (NHA), but no correlation was observed between TOP1 or TDP1 levels and irinotecan sensitivity. We examined the relative activities of TDP1 and TOP1 along with protein levels with respect to drug responses. Our results show that the TDP1/TOP1 activity ratio has a strong correlation with irinotecan sensitivity. Furthermore, increased TDP1 activity by ectopic expression of TDP1 in GBM cells conferred increased irinotecan resistance.

## 2. Results

### 2.1. Comparison of TOP1 and TDP1 Expression in GBM Cell Lines and Correlation with Irinotecan IC_50_

**Figure 2:**
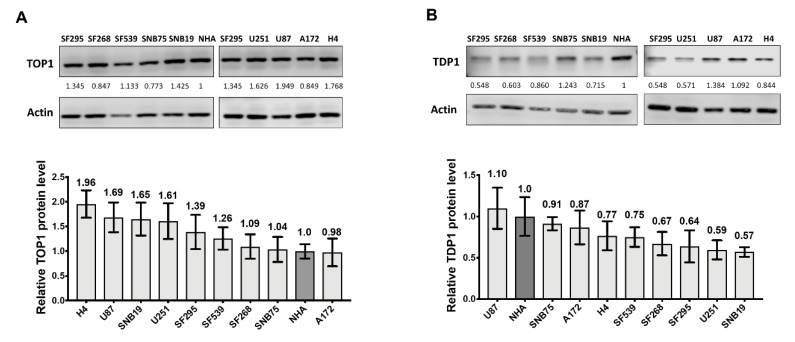
TOP1 and TDP1 protein levels in glioblastoma (GBM) cell lines. Relative levels of (**A**) TOP1 and (**B**) TDP1 protein expression in GBM cell lines were compared by Western blotting against normal human astrocyte (NHA). The signal from actin was used to control the loading of whole cell extract (WCE) proteins (10 µg). Normalized intensity ratios are given for each band, with the intensity ratio of NHA set as 1. The bar graphs show the average and standard deviation as the error bar of measurements from at least three technical replicates in one representative experiment. Whole blots with molecular weight markers are shown in Figure S1.

We compared the TOP1 and TDP1 protein expression level in GBM cell lines by Western blot analysis of whole cell extracts (WCEs). Six cell lines from the National Cancer Institute (NCI-60) panel (SF295, SF268, SF539, SNB75, SNB19, and U251), and three cell lines from the American Type Culture Collection (ATCC) panel (H4, U87, and A172) were included with normal human astrocytes (NHA). We were able to detect both TOP1 and TDP1 in the established cell lines, and the GBM cell lines possessed higher levels of TOP1 protein than NHA (Figure 2A), with relative ratios >1 (Table S1). In contrast, established GBM cell lines had lower levels of TDP1 than NHA (Figure 2B), with relative ratios <1 (Table S1). The IC_50_s for individual cell lines towards irinotecan was measured using a cell-based assay to detect cell abundance (Table 1). No significant correlation (R = −0.254) was found between TOP1 protein levels and irinotecan IC_50_s (Table S2). However, a weak correlation between higher TDP1 protein levels and increased IC_50_ values was observed (R = 0.797, *p* = 0.01; Table S2). These trends held across three distinct biological replicates (Figure S2, Table S2), suggesting that TOP1 and TDP1 levels alone may not be highly predictive indicators of irinotecan vulnerability.

### 2.2. TOP1 Enzymatic Activity in GBM Cell Lines Does not Correspond to the TOP1 Protein Level

**Figure 3:**
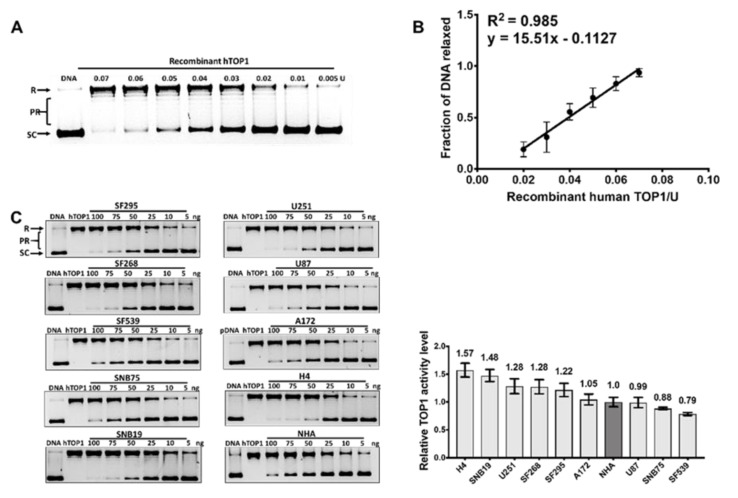
Topoisomerase I (TOP1) activity in GBM cell lines. (**A**) Supercoiled plasmid DNA was relaxed by serially diluted recombinant human topoisomerase I (hTOP1) and separated using gel electrophoresis. SC: supercoiled DNA, R: relaxed DNA, PR: partially relaxed DNA; (**B**) the fraction of DNA relaxed by the TOP1 activity was quantitated to generate a standard curve; (**C**) assay of relative TOP1 activity levels in serial dilutions of GBM WCEs. The bar graph shows the average and standard deviation as the error bar from at least three technical replicates.

TOP1 relaxation activity can be measured using supercoiled plasmid DNA as a substrate [13]. Human TOP1 belongs to the type IB topoisomerase family and does not require ATP or divalent metal ions for its catalytic activity. Recombinant human TOP1 (hTOP1) protein activity (Figure 3A) was used to generate a linear standard curve for the increase in the fraction of DNA relaxed with the increasing level of TOP1 activity (Figure 3B). The TOP1 relaxation activity in each GBM WCE was assayed with serial dilutions of the WCEs (Figure 3C). The quantified values are shown in Table S3. Results from additional biological replicates can be found in Figure S3. The relative TOP1 activity level did not correlate with TOP1 protein concentrations (R = 0.446, Figure S3 and Table S4), suggesting that TOP1 activity in GBM might be modulated by other means. Also, cell specific TOP1 activities did not correlate with irinotecan IC_50_ (Table S2). These studies indicate that TOP1 activity cannot predict irinotecan toxicity in established GBM cell lines.

### 2.3. Measurement of TDP1 Activity in GBM Cell Lines by Gel-Based and Fluorescence-Based Assays

**Figure 4:**
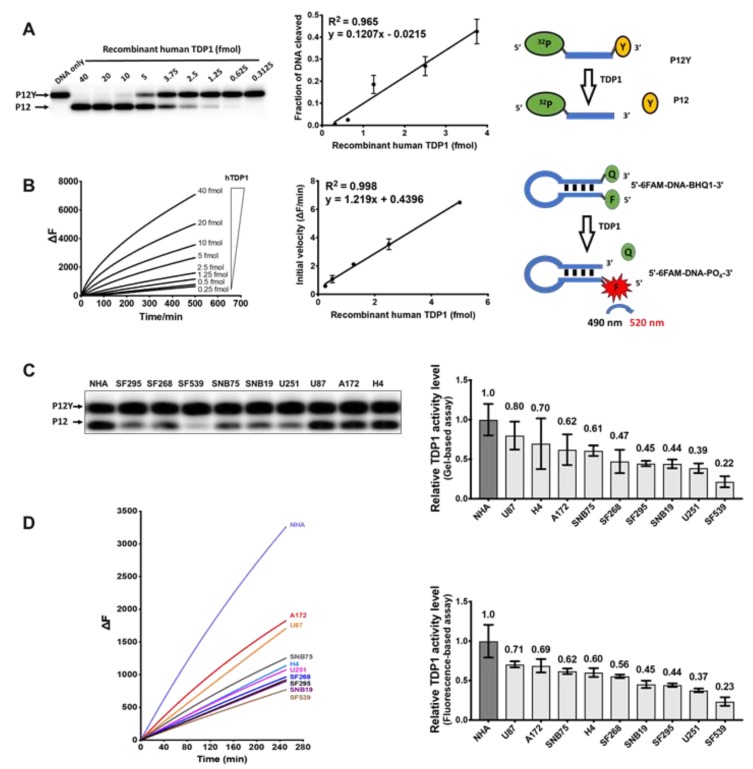
TDP1 activity in GBM cell lines. (**A**) Gel assay for the conversion of radiolabeled P12Y DNA substrate into P12 product by recombinant human TDP1. The standard curve for the fraction of DNA substrate cleaved by increasing amounts of TDP1 is shown on the right; (**B**) TDP1 3’ endonuclease activity on hairpin substrate with 5’ fluorophore and 3’ quencher leads to increase in fluorescence. The initial velocity of fluorescence increase was used to generate a standard curve; (**C**) gel-based assay of relative TDP1 activity levels in GBM WCEs; (**D**) fluorescence-based assay of relative TDP1 activity levels in GBM WCEs. The bar graphs show the average and standard deviation as the error bar (n = 3 or more).

We employed two approaches to measure TDP1 activity in GBM WCEs. Previous studies have demonstrated that human TDP1 is an ATP- as well as a divalent ion-independent enzyme that can hydrolyze the phosphotyrosyl-linkage at the 3’ end of DNA without sequence specificity [22,24]. In addition, TDP1 can act as a broad-spectrum 3’ exonuclease which is able to hydrolyze various phosphodiester linkages at the 3’ ends of DNA [24,32]. The TDP1 activity is efficient on single-stranded or blunt-ended oligonucleotides [24,32]. To determine TDP1 activity, we used a gel-based assay with a 5’-^32^P labeled single-stranded oligonucleotide with a 3’ tyrosine modification (P12Y) as the substrate to yield P12 as the TDP1 reaction product in proportion to an increasing amount of recombinant human TDP1 (Figure 4A). In an alternative fluorescence-based assay, a hairpin-structured blunt-ended oligonucleotide (Figure 4B) with a 5’ fluorophore and a 3’ BHQ1 modification was used as a substrate [33]. TDP1 removal of the quencher at the 3’-end of the substrate led to an increase in fluorescence signal (ΔF). Activities in the GBM WCEs, measured in triplicates by the gel-based assay (Figure 4C) or fluorescence-based assay (Figure 4D), were calculated based on the standard curve of recombinant human TDP1 (hTDP1) (Figure 4A,B) and normalized to TDP1 activity level in NHA (Table S5). Results from additional replicates can be found in Figure S4. The TDP1 activities obtained from the gel-based and fluorescence-based assays showed strong correlations (R = 0.943, Figure S4, panel D4), demonstrating their similar capabilities for measuring TDP1 activities in vitro. EDTA was present in both assay buffers to suppress the metal ion-dependent exonuclease activities present in the WCEs. TDP1 activity levels measured by the two assay methods correlate only slightly with TDP1 protein levels measured by Western blot (Table S4) and with irinotecan IC_50_s (Table S2).

### 2.4. TDP1/TOP1 Activity Ratio in GBM WCE Is a Strong Predictor of Irinotecan IC_50_

**Figure 5:**
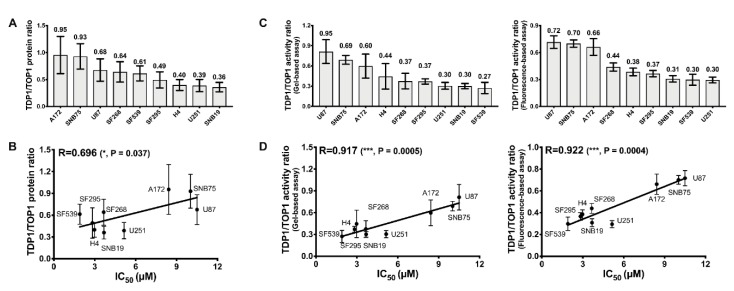
TDP1/TOP1 activity ratio is a strong predictor for irinotecan IC_50_. (**A**) TDP1/TOP1 protein ratio levels and (**B**) Pearson correlation between the TDP1/TOP1 protein ratio and irinotecan IC_50_ for GBM cell lines; (**C**) TDP1/TOP1 activity ratio levels in GBM WCEs and (**D**) Pearson correlation between irinotecan IC_50_ and the TDP1/TOP1 activity ratio based on TDP1 activity, measured by both gel-based and fluorescence-based assays.

Previous studies have demonstrated that TOP1 expression is necessary for cell cytotoxicity to TOP1 poisons and that loss of TDP1 activity would lead to hypersensitivity to TOP1 poisons [13,22]. However, our results above indicate that TOP1 and TDP1 protein expression levels in GBM could not accurately predict the TOP1 and TDP1 activity levels, and that the protein level or activity level of TOP1 and TDP1 individually are not useful indicators of the GBM cellular response to irinotecan. The lack of correlation between TOP1 and TDP1 protein expression (Table S6) suggests that there is no overlapping pathway for regulating TOP1 and TDP1 levels. To further identify more promising indicators, we calculated the TDP1/TOP1 protein level ratio and the TDP1/TOP1 activity level ratio (Table S7). The TDP1/TOP1 protein ratios (Figure 5A) were weakly correlated with irinotecan IC_50_ (R = 0.696, *p* = 0.037; Figure 5B). However, the TDP1/TOP1 activity ratios (Figure 5C) were strongly correlated with the irinotecan IC_50_ values (R = 0.917, *p* = 0.0005 for gel based TDP1 assay; R = 0.922, *p* = 0.0004 for fluorescence based TDP1 assay; Figure 5D). The results from the other two sets of experiments with the GBM cell lines also confirmed that the TDP1/TOP1 activity ratio is a strong predictor for irinotecan IC_50_ (Figure S5, Table S2), with Pearson correlation coefficient R = 0.972 for the average of the gel-based TDP1/TOP1 activity ratios from all three sets of experiments.

### 2.5. Increased Resistance to Irinotecan Following Transfection with Recombinant TDP1

**Figure 6:**
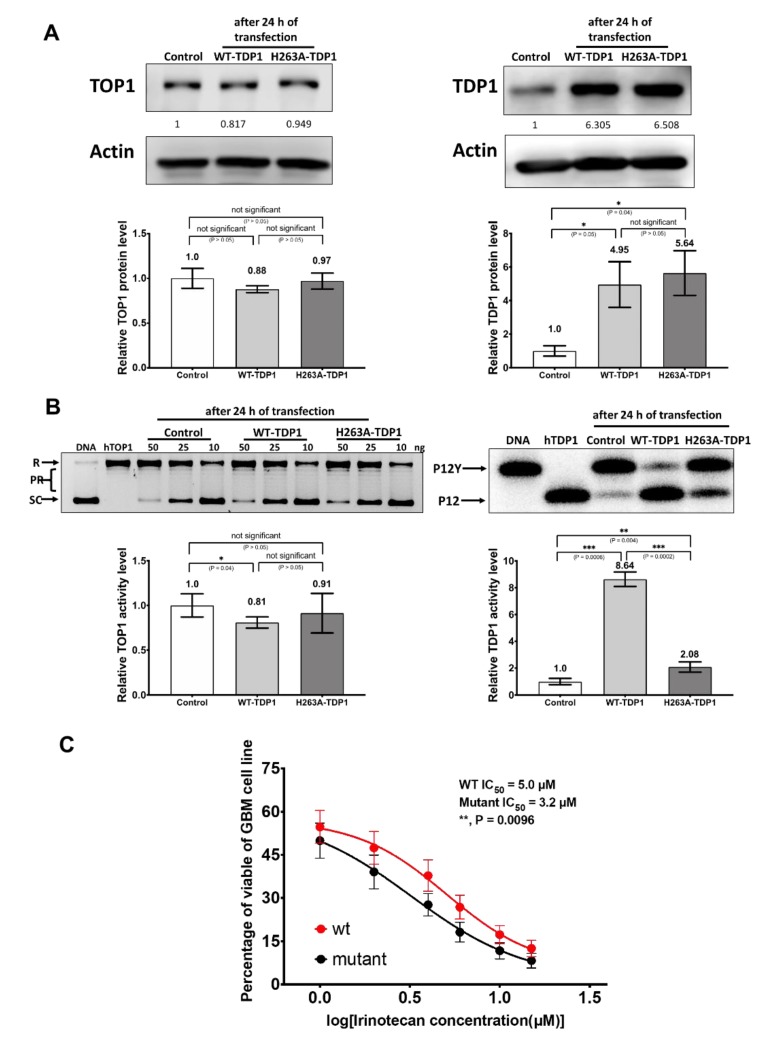
Transfection of TDP1 into the H4 cell line to elevate the TDP1/TOP1 activity ratio and increase the irinotecan IC_50_. (**A**) TDP1 and TOP1 protein levels after transfection of H4 with wild type (WT-TDP1) or mutant (H263A-TDP1) clones. Cells were collected 24 h after transfection and 10 µg of WCEs were analyzed by western blots. Whole blots with molecular weight markers are shown in Figure S6. The signal from actin was used to control the loading of WCE proteins. Normalized intensity ratios are given for each band, with the intensity ratio of mock-treated control set as 1. The bar graphs show the average and standard deviation of measurements as error bars from all technical replicates (n = 3 or more); (**B**) TDP1 and TOP1 activity levels in H4 WCE from the transfection of WT-TDP1. Cells were collected 24 h post-transfection and 4 µg of WCE was used in the gel-based assay for TDP1 activity; (**C**) Irinotecan IC_50_ for H4 cells transfected with the WT-TDP1 clone versus H263A mutant TDP1 clone (n = 7).

Previous study has already shown that the knockdown of TDP1 activity by siRNA in rhabdomyosarcoma cell lines or by shRNA in lung fibroblast cell line increases the sensitivity to CPT [29,34]. Furthermore, TOP1 depletion by siRNA was found to sensitize colorectal cancer cell lines to irinotecan treatment in a TOP1-dependent manner [28]. To follow up on our observed correlation between irinotecan sensitivity and the TDP1/TOP1 activity ratio in GBM cell lines, we elevated the TDP1 activity in H4 cell line by transfection with clones expressing wild-type (WT)-TDP1 or null activity mutant H263A-TDP1 [19] to determine the effect on irinotecan IC_50_. The overexpression of TDP1 and unchanged expression of TOP1 were confirmed by Western blotting (Figure 6A). An increase in TDP1 catalytic activity in the WCE from WT-TDP1 but not H263A-TDP1 was also observed (Figure 6B), along with no change in TOP1 catalytic activity, as expected. H4 cells transfected with WT-TDP1 had significantly higher Irinotecan IC_50_ than H4 cells transfected with the H236A-TDP1 clone (Figure 6C). This result further supports the correlation between the TDP1/TOP1 activity ratio and Irinotecan IC_50_ for GBM.

### 2.6. Carboxylesterase 2 (CES2) Activity in the GBM Cell Lines

**Figure 7:**
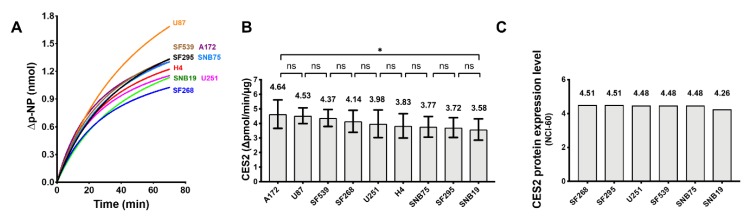
Comparison of carboxylesterase 2 (CES2) activity in the WCE of GBM cell lines. (**A**) The time course of p-NP (p-nitrophenol) generation attributable to CES2, calculated by subtracting the p-NP signal obtained in the presence of the CES2 inhibitor loperamide; (**B**) the variation in CES2 activity in GBM cell lines measured over the initial 20 min of reaction. Results represent the average from nine replicate measurements in two experiments; (**C**) The CES2 protein expression level based on SWATH-MS (Sequential Windowed Acquisition of All Theoretical Fragment Ion Mass Spectra) from the CellMinerCDB database for the GBM cell lines in the NCI-60 panel.

Irinotecan is converted to the much more potent metabolite SN-38 by carboxylesterase isoform CES2 [35]. The CES2 activity present in the GBM cell lines studied here were assayed and found to be comparable (Figure 7). This demonstrates that the relatively higher level of resistance of cell lines such as A172 and SNB75 to irinotecan was not due to lack of CES2 activity in these cells, and the irinotecan sensitivity of SNB19 was not due to a relatively high level of CES2 being present in SNB19. Metabolic conversion of irinotecan to SN-38 could be a significant factor for irinotecan treatment efficacy in vivo [36]. Nevertheless, the observation made here that GBM cells with a lower TDP1/TOP1 activity ratio would be more sensitive to the TOP1 poison inhibitor is valid with the GBM CES activity data being taken into consideration.

### 2.7. Variable Levels of TDP1, TOP1 Activities, and the TDP1/TOP1 Activity Ratio in GBM Patient Tumor Cell Lysates

**Figure 8:**
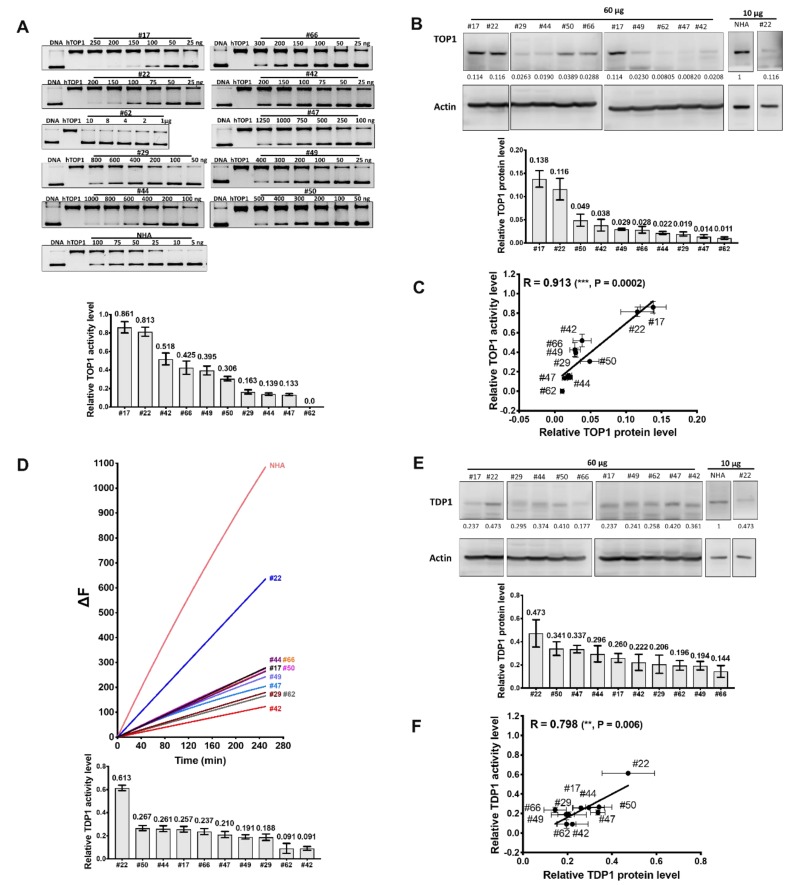
TOP1 and TDP1 activity and protein levels in GBM patient tumor WCEs. (**A**) Assay of TOP1 activity in GBM patient tumor WCEs; (**B**) Western blot analysis of TOP1 protein expression. The first two panels are from the same blot. Signal from actin was used to control loading of WCE proteins (60 µg). Normalized intensity ratios are given for each band, with the intensity ratio of NHA set as 1; (**C**) Pearson correlation of relative TOP1 protein and activity levels in GBM patient tumor WCEs; (**D**) fluorescence-based assay of TDP1 activity; (**E**) Western blot analysis of TDP1 protein expression; (**F**) Pearson correlation of relative TDP1 protein and activity levels in GBM patient tumor WCEs. The bar graphs (n = 3 or more) show the average and standard deviation as error bars for relative TOP1 and TDP1 activity or protein levels normalized against activity and protein expression in NHA. Whole blots with molecular weight markers are shown in Figure S7.

**Figure 9:**
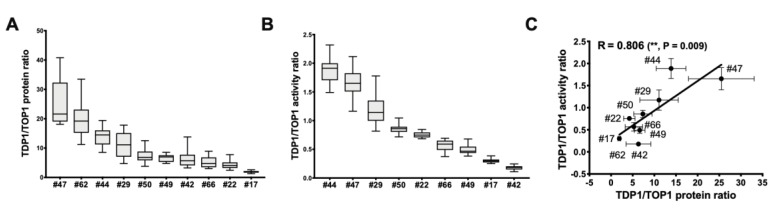
Range of TDP1/TOP1 protein ratio and activity ratios in GBM patient tumor WCEs. The box plots show the median, with minimum and maximum values at the bottom and top tail-ends for (**A**) TDP1/TOP1 protein ratio; (**B**) TDP1/TOP1 activity ratio calculated from the relative TOP1, TDP1 protein and activity levels of each GBM patient tumor WCE; (**C**) Pearson correlation between the TDP1/TOP1 protein ratio and TDP1/TOP1 activity ratio.

TOP1 and TDP1 expression, as well as activity in the WCEs of ten GBM tumors from patients (de-identified data shown in Table S8), were measured (Figure 8). The results (Table S9) showed that the GBM patient tumor WCEs exhibited a greater range of TOP1 expression and activity than TDP1. The range of TDP1/TOP1 protein and activity ratio is shown in Figure 9. It should be noted that tumor #62 is not included in the graph for TDP1/TOP1 activity ratio in Figure 9B because of the extremely low TOP1 activity in the WCE of this tumor specimen. The results show a better correlation between the TOP1, TDP1 activity levels and TOP1, TDP1 protein levels for the GBM patient tumors than the GBM cell line (Table S4). GBM patients with a relatively high or low TDP1/TOP1 protein or activity ratio in the tumors could potentially be identified in the process of chemotherapy treatment selection.

## 3. Discussion

GBM is a devastating disease with poor prognosis and a lack of predictive biomarkers for chemotherapy [1]. The prodrug irinotecan is converted to a topoisomerase I poison specifically to stabilize the TOP1cc on chromosomal DNA. Irinotecan has been found in clinical trials to exhibit modest and not consistently reproducible efficacy in the treatment of GBM. Therefore, our study is aimed at evaluating the roles of TOP1 as a target and the TOP1cc repair enzyme TDP1 on the sensitivity of GBM to irinotecan treatment in order to identify rational predictive biomarkers that may contribute to improving the treatment outcomes.

Previous studies have shown that the cytotoxic activity of TOP1 poisons is associated with TOP1 and TDP1 protein expression, as well as their catalytic activities. The infection of GBM cell lines with adenovirus Delta-24 has been shown to increase the expression and activity of TOP1 and enhances the antiglioma effect of irinotecan [37]. A preliminary study on nine clinical colorectal tumors indicated that the three samples with the highest TOP1 expression and activity were from patients who responded to irinotecan treatment [38]. Although a later study on the roles of TDP1 and TOP1 in modulating colorectal cancer response to irinotecan demonstrated that TDP1 overexpression or TOP1 depletion is protective, while conversely TDP1 depletion leads to TOP1-dependent hypersensitivity to irinotecan, there was no correlation between inherent TDP1 or TOP1 protein levels alone and irinotecan sensitivity [28]. A study on small cell lung cancer (SCLC) cell lines found that TDP1/TOP1 protein ratio is a better predictor than the individual TDP1 or TOP1 protein level, showing good correlation with irinotecan sensitivity in eight out of ten cell lines examined [39].

In our study, we found that the TDP1/TOP1 activity ratio was superior to TDP1/TOP1 protein ratio as a predictor for the response of GBM cell lines to irinotecan treatment, as indicated by the irinotecan IC_50_s measured here. This may be due to post-translational modification (PTM) of TOP1 and TDP1 proteins in GBM. Previous studies have shown that PTMs can have a significant influence on TOP1 activity and drug cytotoxicity. TOP1 phosphorylation, at the PT268 or PS506 site, increases TOP1 activities and cellular sensitivity to CPT-induced apoptosis [31,40]. O-GlcNAcylation is also found to contribute to the activity elevation of TOP1 [41]. We suggest that the PTMs confer TOP1 activity alterations and might account for the weaker correlation between the TOP1 protein and its activity levels in GBM cell lines. A previous study [42] reported enhanced MKP-1 phosphatase activity in GBM cells, including U251, studied here. Knockdown of the MKP-1 was found to enhance cell death caused by cancer drugs. The effect on irinotecan-induced cell death was more prominent than other drugs. It is possible that MKP-1 phosphatase activity produces a higher TDP1/TOP1 activity ratio by removing a phosphorylation on TOP1 that can activate the TOP1 activity. Breast cancer cell lines selected for resistance following exposure to irinotecan have been found to have TOP1 with altered isoelectric points, consistent with altered PTMs of TOP1 [43]. In contrast, the lesser influence of PTM on TDP1 activity might account for the stronger correlation between the TDP1 protein and its activity level. In addition to z-scores for irinotecan, drug sensitivity z-scores for camptothecin and topotecan are also available in the CellMiner database for the six GBM cell lines included in the NCI-60 panel (Table S10) [44,45]. The sensitivity z-scores for these TOP1 inhibitors all show better correlation with our TDP1/TOP1 activity ratio than the TDP1/TOP1 protein ratio or TDP1 protein and activity level alone (Table S11). As expected, there was little or no correlation between TDP1/TOP1 activity ratio and sensitivity z-scores for TOP2 inhibitors in the CellMiner database (Table S11).

The low expression of TDP1 observed in the GBM cell lines may reflect repair pathway deficiencies in cancer development. The increase of irinotecan IC_50_ resulting from the transfection of the GBM H4 cell line with the recombinant TDP1 clone is consistent with TDP1 activity being an important determinant of GBM sensitivity to irinotecan treatment. A decrease in TDP1 activity in the U87 cell, by either knockdown or inhibitor, would be expected to decrease the IC_50_ of this relatively resistant GBM cell line, as demonstrated previously for other cancer cell lines [28,29,34]. Combination therapy that targets both TOP1 and TDP1 has great potential to improve the treatment outcomes of GBM. In addition, TDP1 possesses the versatility of precisely hydrolyzing a variety of 3’ adducts from DNA [22,32,46,47], and therefore, the screening of TDP1 inhibitors is a promising avenue for developing new cancer therapies [48,49,50,51]. 

There are additional cellular factors that contribute to irinotecan treatment outcome. The CPT-induced degradation of TOP1, via the efficient degradation of TOP1cc, is necessary for sufficient exposure of the phosphodiester linkage to TDP1 hydrolysis [13,52,53]. Previous studies have shown that DNA damage-induced PTMs are associated with TDP1 N-terminal domain (NTD), a dispensable domain for TDP1 catalytic activity (residue 1–148). Phosphorylation, SUMOylation, and PARylation at NTD stabilize TDP1 and promote TDP1 recruitment on the DNA damage site without interfering with its activity [54,55,56,57].

It is worth noting that drug sensitivities are also determined in part by alternative repair pathways and epigenetic modifications. PNKP and XRCC1 are essential enzymes for single-strand break repair following the TDP1 repair process [13]. RECQ1, Mus81-EME1, and XPF-ERCC1 could provide resistance to CPT-induced apoptosis on either single-strand break repair or double-strand break repair pathways [58,59,60]. The PARylation of TOP1 reduces its activity in vivo by regulating TOP1 delocalizing from the nucleolus to nucleoplasm and increases cell resistance [61]. In addition, perturbed histone acetylation stimulates faster access by DNA repair enzymes to the damaged sites and subsequently provokes a higher resistance [62], and may have to be considered for the optimization of GBM treatment in the future.

## 4. Materials and Methods

### 4.1. GBM Cell Lines

The GBM cell lines U87, A172, and H4 were obtained from American Type Culture Collection (ATCC). Cell lines SF295, SF268, SF539, SNB75, SNB19, and U251 were provided by National Cancer Institute Division of Cancer Treatment and Diagnosis (NCI, DCTD). Cell lines were cultured and maintained in Dulbecco’s Modified Eagle’s medium (DMEM, Corning Inc., Corning, NY, USA) supplemented with 10% heat-inactivated fetal bovine serum (Hyclone FBS, Thermo Fisher Scientific, Waltham, MA, USA), 1% Penicillin-Streptomycin (Thermo Fisher Scientific), and 0.1% Plasmocin Prophylactic (InvivoGen, San Diego, CA, USA). All cells were grown at 37 °C in a humidified incubator with 5% CO_2_. Human astrocytes were purchased from ScienCell Research Laboratories (Carlsbad, CA, USA) and cultivated in accordance with the manufacturer’s protocol, using Astrocyte Medium (ScienCell) on poly-L-lysine-coated cell culture flasks and dishes. Cells were used for only two subcultures to prevent the analysis of senescent cells.

### 4.2. GBM Whole Cell Extract (WCE) Preparation

To prepare WCEs for the analysis of proteins, cells were plated at a density of 4 × 10^5^ cells in 60 mm dishes and collected until 80% confluence. Briefly, cells were washed twice in 1 × phosphate-buffered saline (Gibco PBS, Thermo Fisher Scientific) and lysed in radioimmunoprecipitation assay buffer (RIPA, 50 mM Tris-HCl, pH 7.4, 150 mM NaCl, 5 mM EDTA, 1 mM EGTA, 1% NP-40, 0.1% SDS, and 0.5% sodium deoxycholate) supplemented with 1 mM phenylmethanesulfonyl fluoride (PMSF), 1% (*v*/*v*) of Halt Protease inhibitor cocktails (Thermo Fisher Scientific), and 1% (*v*/*v*) Halt Phosphatase inhibitor cocktails (Thermo Fisher Scientific). Cells were incubated with lysis buffer on ice for 5 min and then transferred to a sterile microcentrifuge tube, followed by 30%, 30 s sonication with a Thermo Fisher Scientific 120 W microtip sonicator. The cell debris was removed by centrifugation at 15,000× *g* at 4 °C for 15 min and the supernatant protein concentration was measured using the Pierce BCA Assay Kit (Thermo Fisher Scientific).

### 4.3. Western Blots

Protein expression levels were detected using Western blots. Briefly, proteins from whole cell extracts (WCE) were first separated by 7.5% SDS-PAGE and then transferred to nitrocellulose membrane with a transfer buffer (48 mM Tris, 39 mM glycine, 0.4% SDS, 20% *v*/*v* methanol) at 100 V for 1 h. The membrane was blocked with 5% bovine serum albumin (BSA) in Tris-buffered saline (TBS) at room temperature for 1 h, followed by incubation of 1:1000 (*v*/*v*) primary antibody diluted in 1% Tween 20 in TBS (TBST) at 4 °C for 18 h. The membrane was washed three times for 5 min (3 × 5 min) and the horse-radish peroxidase (HRP) conjugated secondary antibody 1:5000 (*v*/*v*) diluted in TBST was incubated with the membrane at room temperature (RT) for an additional 1 h. The membrane was then washed 3 × 5 min before the target protein expression level was developed with SuperSignal West Pico Plus Chemiluminescent Substrate (Thermo Fisher) for 5 min. The images were obtained with a C-DiGit Blot scanner (LI-COR, Lincoln, NE, USA) and the expression density from the target protein was analyzed by ImageStudio (LI-COR). The information on primary and secondary antibodies is listed in Table S12. The Western blot TOP1 or TDP1 signal intensity for each GBM cell line WCE was first normalized to actin signal to correct for loading, then divided by the NHA TOP1 or TDP1 signal intensity for the comparison of relative TOP1 or TDP1 protein levels. GraphPad Prism version 8.1 was used to carry out the data analysis. The mean and standard deviations shown in bar graphs were calculated based on at least three technical replicates. The degree of correlation between the two parameters tested was determined by using the Pearson correlation coefficient value (R) and considered as significant for *p* < 0.05. Each entire set of experiments was repeated three times.

### 4.4. Irinotecan IC_50_ Measurement for GBM Cell Lines

For IC_50_ measurement of GBM cell lines, cells were plated at a density of 8.5 × 10^3^ cells/well in a 96-well plate and cultured until 70–80% confluence. Concentrated irinotecan dissolved in DMSO was serially diluted (0.05 to 15 µM) in freshly prepared culture media, which were used for further 72 h incubation of the cells. The cell viabilities were assayed by TO-PRO-3 Stain (Thermo Fisher Scientific) according to the manufacturer’s protocol. Briefly, cells were first fixed using 100 µL/well of 4% paraformaldehyde/PBS (PFA) for 20 min at RT and permeated with 0.2% TritonX-100/PBS by a further 30 min of rocking. Cells were then blocked by the blocking buffer (LI-COR) for 30 min and stained by adding diluted TO-PRO3 reagent at a ratio of 1:1000 in blocking buffer for an additional 1 h rocking. Before reading, cells were rinsed four times with 0.1% Tween/PBS. The far-red fluorescence signal from the TO-PRO3 reagent was detectable by the Odyssey CLx Imaging System (LI-COR) from surviving cells, which were analyzed by the ImageStudio system (LI-COR). IC_50_ was determined as the concentration of irinotecan which results in a 50% of cell viability compared to the DMSO control. Results were obtained from six technical replicates conducted in each of five biological replicates.

### 4.5. Human Topoisomerase I (hTOP1) Relaxation Activity Measurement 

Negatively supercoiled pBAD/Thio plasmid DNA (240 ng) purified by cesium chloride gradient centrifugation was used as the substrate for the hTOP1 relaxation assay. The relaxation assay was conducted for established GBM cell lines and NHA WCEs in a reaction buffer supplemented with EDTA to chelate with metal ions and thereby suppress the activities from the metal ion-dependent type IA and type IIA topoisomerase activities present in the WCE. The reaction was carried out in a volume of 20 µL in a reaction buffer of 10 mM Tris-HCl, pH 7.9, 1 mM EDTA, 150 mM NaCl, 0.1% BSA, 0.1 mM spermidine, and 5% glycerol. After 30 min at 37 °C, reactions were stopped by adding 4 µL of 6× SDS stop buffer (6% SDS, 0.3% bromophenol blue, 30% glycerol). Supercoiled substrate DNA and relaxed DNA products were separated based on difference in electrophoretic mobility in 1% agarose gel. The DNA molecules in the gel were stained with 1 µg/mL of EtBr solution and photographed over UV light. AlphaView SA) were used to generate a reference standard curve for the fraction of supercoiled DNA converted to relaxed DNA by units (U) of hTOP1 as defined by the supplier. TOP1 activity in whole cell extracts (WCEs) was assayed under the same conditions. Serial dilutions of WCEs were assayed to identify the amount needed to relax 50% of the supercoiled DNA substrate and calculate the hTOP1 activity present in each WCE based on the standard curve as U/µg of total WCE protein. The relative hTOP1 activity levels present in the cell lines were normalized by the hTOP1 activity level in fetal normal human astrocyte (NHA).

### 4.6. Human TDP1 Activity Measurement

#### 4.6.1. Gel-Based TDP1 Assay

The 12-base long oligonucleotide substrate linked to tyrosine at the 3’-end (5’-HO-GAAAAAAGAGTT-PO4-Tyr-3’) was obtained from TopoGEN and labeled at the 5’-end with (γ-^32^P) ATP and T4 polynucleotide kinase (NEB). The radiolabeled oligonucleotide substrate P12Y was purified by centrifugation through a 1 mL Sephadex G15 column and stored in 10 mM Tris-HCl pH 7.5 at −20 °C until further use. Recombinant human tyrosyl-DNA phosphodiesterase 1 (hTDP1) was purified as described [63]. Serial dilutions of hTDP1 were incubated with P12Y oligo (4 ng) at 37 °C for 30 min in 5 µL of reaction buffer (20 mM Tris-HCl, pH 7.5, 100 mM KCl, 10 mM EDTA, 1 mM DTT). The reaction was stopped by adding 5 µL of 2 × stop solution (96% formamide, 20 mM EDTA, 0.03% xylene cyanol and 0.03% bromophenol blue) followed by heat-inactivation at 95 °C for 5 min. Oligonucleotide P12 Y substrate and P12 product with 3’-tyrosine removed by hTDP1 were separated by electrophoresis in 20% urea-denaturing sequencing gel. Following analysis with the BioRad Pharos FX Plus Phosphorimager, the correlation between hTDP1 activity, represented as fmol, and the fraction of P12 produced was plotted as a standard curve. TDP1 activity assay for WCE proteins was carried out under the same conditions, with 2 µg of WCE proteins added to each reaction. The TDP1 activity present in each microgram of WCE was calculated based on the standard curve as fmol/µg. The TDP1 activities in the GBM cell lines were normalized by TDP1 activity in NHA. 

#### 4.6.2. Human TDP1 Activity Measurement by Fluorescence-Based Assay

Human TDP1 (hTDP1) activity was also carried out by fluorescence-based assay as described [33]. The 5’ phosphorothioate bond linked fluorophore, 6-FAM, and 3’ phosphodiester bond linked quencher, BHQ1, were modifications present on a 55 nucleotides long 5’FAM-DNA-BHQ1-3’ oligonucleotide substrate (5’-6-FAM-AAA GCA GGC TTC AAC GCA ACT GTG AAG ATC GCT TGG GTG CGT TGA AGC CTG CTT T-BHQ1-3’, purchased from LGC BioSearch) that formed a hairpin structure. Briefly, 25 pmol of 5’FAM-DNA-BHQ1-3’ was incubated with serial dilutions of hTDP1 at 37 °C in a reaction buffer of 20 mM Tris-HCl, pH 8.0, 100 mM KCl, 10 mM EDTA, 10 mM DTT, 0.05% TritonX-100, with a final volume of 25 µL. The fluorescence signal from each reaction was measured every 30 s for 500 min. The correlation between the amount of hTDP1, present as fmol, and the initial linear slope (0–125 min) from each reaction was plotted as a standard curve. TDP1 activity present 2 µg of WCE was assayed under the same conditions and calculated based on the standard curve as fmol/µg WCE. The relative TDP1 activity levels in the cell lines were normalized by the TDP1 activity level in NHA.

### 4.7. Transfection with Wild-Type (WT) and Mutant TDP1 cDNA Clones

The TDP1 cDNA clone with expression under the control of the CMV promoter in pCMV6-XL4 was obtained from OriGene. H263A substitution was performed using the Q5 Site-Directed Mutagenesis Kit (NEB) with 5’-TGGAACACACGCCACGAAAATGATG-3’ and 5’-AACGCAATATCCAACTTTG-3’ primers to generate a mutant with null activity [24]. GBM H4 cells were seeded at a density of 1.8 × 10^5^ cells/well in 35 mm dishes and cultured 24 h until 60% confluence. DNA (500 ng) was mixed with Lipofectamine 3000 (Invitrogen) at a ratio of 1:2 (w/v) in 250 µL of Opti-MEM Medium (Gibco) for 20 min at RT and added to cells. After further incubation for 24 h, WCEs were prepared using the RIPA buffer, as described above. For measurement of irinotecan IC_50_, H4 cells were plated at a density of 6.5 × 10^3^ cells/well and cultured for 24 h until 60% confluence. DNA was mixed with Lipofectamine 3000 in 10 µL of Opti-MEM Medium for 20 min at RT and incubated with cells for 24 h before the addition of serial dilutions of irinotecan. After further incubation for 72 h, cell viabilities were measured with the TO-PRO3 assay, as described above.

### 4.8. Protein Extraction from GBM Patient Samples

Glioblastoma tumors were collected from the patients during operations at the Miami Neuroscience Center of Larkin Hospital according to the protocol approved by Florida International University Institutional Review Board (No. IRB-16-0355-CR02), and snap-frozen in cryotubes pre-cooled on dry ice. Samples kept in dry ice were then delivered by courier to Florida International University and cryopreserved in liquid nitrogen. Ten GBM tumors were used to prepare WCEs. Frozen tumor tissues were transferred onto an ice-cooled 100 mm dish and sliced into small pieces. The tumor segments were then weighed and the RIPA/inhibitors lysis buffer was added at a ratio of 1:10 (v/w). Samples were homogenized by Dounce Homogenizer and rotated at 4 °C for 2 h, followed by centrifugation at 15,000× *g* for 15 min. Supernatant was kept in aliquots at –80 °C for further analysis.

### 4.9. Carboxylesterase 2 Activity Measurement

The carboxylesterase 2 (CES2) activity in each cell line was assayed as described [64] using 6.7 µg of WCE proteins in the presence or absence of 300 µM of CES2 inhibitor loperamide (MilliporeSigma, St. Louis, MO, USA) in 20 µL of 100 mM Tris-HCl, pH 7.4 pre-incubated at 37 °C for 10 min. The reaction was initiated by adding 200 µL of p-NPA (4-Nitrophenyl acetate, MilliporeSigma) substrate. Absorbance at 405 nm was recorded at 0, 5, 10, 20, 30, 40, 50, and 60 min. The amount of the reaction product p-NP formed was calculated based on the standard curve from serial dilutions of p-NP (MilliporeSigma). The carboxylesterase activity from each microgram of WCE was represented as pmol/min/µg. The difference in activity obtained in the presence and absence of loperamide was considered CES2 activity. The assay was performed in a total of nine replicates in two experiments.

### 4.10. Statistical Analysis

The correlation between two parameters was determined using the Pearson correlation coefficient value (R) and considered significant when the *p*-value was less than 0.05. The significance of the difference between the two groups was determined by the pairwise t-test (not significant, *p*-value > 0.05; *, *p*-value 0.01 to 0.05; **, *p* -value 0.001 to 0.01; ***, *p* -value 0.0001 to 0.001; ****, *p* -value < 0.0001). The bar-graph is presented as average ± standard deviation (SD) as the error bar. GraphPad Prism version 8.1 was used to carry out the data analysis.

## 5. Conclusions

This study demonstrates that the variation in TDP1/TOP1 activity ratio in GBM could be a potential predictive biomarker for irinotecan treatment. Further study on TDP1 and TOP1 protein expression and activity in patient-derived GBM models along with clinical outcome is required. Inhibiting TDP1 activity is a plausible approach to improve irinotecan effectiveness in GBM.

## Figures and Tables

**Table 1:** Glioblastoma cell lines sensitivities to irinotecan treatment.

Cell Lines	Irinotecan IC_50_ (μM)
SF539	1.91 ± 0.32
SF295	2.83 ± 0.51
H4	2.93 ± 0.44
SF268	3.66 ± 0.76
SNB19	3.68 ± 0.55
U251	5.15 ± 1.17
A172	8.42 ± 0.94
SNB75	10.02 ± 1.38
U87	10.50 ± 1.74

The average and standard deviations shown are from six technical replicates conducted in each of the five biological replicates.

## Supplementary Materials

The following are available online at https://www.mdpi.com/2072-6694/11/10/1416/s1, Figure S1: Blots from Figure 2A,B, Figure S2: TOP1 and TDP1 protein levels measurements in different sets of GBM cell lines to assess potential correlation with irinotecan IC_50_, Figure S3: Topoisomerase I (TOP1) activity in replicated sets of GBM cell line experiments does not correlate with TOP1 protein levels or irinotecan IC_50_, Figure S4: TDP1 activity levels show slight correlation with irinotecan IC_50_, Figure S5: TDP1/TOP1 activity ratio is a stronger predictor than TDP1/TOP1 protein ratio for irinotecan IC_50_,_._ Figure S6: Blots from Figure 6A. Figure S7: Blots from Figure 8B and 8E; Table S1: Relative TOP1 and TDP1 protein expression levels in GBM cell lines, Table S2: Pearson correlation of TOP1 and TDP1 protein or activity, TDP1/TOP1 protein or activity ratio with experimental IC_50_ of irinotecan, Table S3: TOP1 activity levels in GBM cell lines, Table S4: Pearson correlation of protein level with activity level of TOP1 and TDP1 in GBM cell lines and tumors, Table S5: TDP1 activity levels in GBM cell lines, Table S6: Pearson correlation between TOP1 levels and TDP1 levels in GBM cell lines and GBM patient tumors, Table S7: TDP1/TOP1 ratios in GBM cell lines, Table S8: Glioblastoma patient information, Table S9: TOP1, TDP1, and TDP1/TOP1 ratio in GBM patient tumors, Table S10: Drug sensitivity z-scores of topoisomerase inhibitors for GBM cell lines in the NCI-60 panel, Table S11: Pearson correlation of TOP1 and TDP1 protein or activity, TDP1/TOP1 protein or activity ratio with z-score of topoisomerase inhibitors, Table S12: Information on primary and secondary antibodies.

## References

[1] Paolillo M., Boselli C., Schinelli S. (2018). Glioblastoma under Siege: An Overview of Current Therapeutic Strategies. Brain Sci..

[2] Fuchs C., Mitchell E.P., Hoff P.M. (2006). Irinotecan in the Treatment of Colorectal Cancer. Cancer Treat. Rev..

[3] Van Meerbeeck J.P., Fennell D.A., De Ruysscher D.K. (2011). Small-Cell Lung Cancer. Lancet.

[4] Goldwirt L., Beccaria K., Carpentier A., Farinotti R., Fernandez C. (2014). Irinotecan and Temozolomide Brain Distribution: A Focus on ABCB1. Cancer Chemother. Pharmacol..

[5] Cloughesy T.F., Filka E., Nelson G., Kabbinavar F., Friedman H., Miller L.L., Elfring G.L. (2002). Irinotecan Treatment for Recurrent Malignant Glioma using an Every-3-Week Regimen. Am. J. Clin. Oncol..

[6] Friedman H.S., Petros W.P., Friedman A.H., Schaaf L.J., Kerby T., Lawyer J., Parry M., Houghton P.J., Lovell S., Rasheed K. (1999). Irinotecan Therapy in Adults with Recurrent or Progressive Malignant Glioma. J. Clin. Oncol..

[7] Vredenburgh J.J., Desjardins A., Reardon D.A., Friedman H.S. (2009). Experience with Irinotecan for the Treatment of Malignant Glioma. Neuro Oncol..

[8] Lee E.Q., McFaline-Figueroa J.R., Cloughesy T.F., Wen P.Y. (2018). Is it Time to Reconsider the Role of Irinotecan for the Treatment of High-Grade Gliomas?. Neuro Oncol..

[9] Gruber M.L., Buster W.P. (2004). Temozolomide in Combination with Irinotecan for Treatment of Recurrent Malignant Glioma. Am. J. Clin. Oncol..

[10] Vredenburgh J.J., Desjardins A., Herndon J.E., Marcello J., Reardon D.A., Quinn J.A., Rich J.N., Sathornsumetee S., Gururangan S., Sampson J. (2007). Bevacizumab Plus Irinotecan in Recurrent Glioblastoma Multiforme. J. Clin. Oncol..

[11] Friedman H.S., Prados M.D., Wen P.Y., Mikkelsen T., Schiff D., Abrey L.E., Yung W.K., Paleologos N., Nicholas M.K., Jensen R. (2009). Bevacizumab Alone and in Combination with Irinotecan in Recurrent Glioblastoma. J. Clin. Oncol..

[12] Pommier Y., Leo E., Zhang H., Marchand C. (2010). DNA Topoisomerases and their Poisoning by Anticancer and Antibacterial Drugs. Chem. Biol..

[13] Pommier Y., Sun Y., Huang S.N., Nitiss J.L. (2016). Roles of Eukaryotic Topoisomerases in Transcription, Replication and Genomic Stability. Nat. Rev. Mol. Cell Biol..

[14] Pommier Y. (2006). Topoisomerase I Inhibitors: Camptothecins and Beyond. Nat. Rev. Cancer.

[15] Huang X., Traganos F., Darzynkiewicz Z. (2003). DNA Damage Induced by DNA Topoisomerase I-and Topoisomerase II-Inhibitors Detected by Histone H2AX Phosphorylation in Relation to the Cell Cycle Phase and Apoptosis. Cell Cycle.

[16] Hsiang Y.H., Lihou M.G., Liu L.F. (1989). Arrest of Replication Forks by Drug-Stabilized Topoisomerase I-DNA Cleavable Complexes as a Mechanism of Cell Killing by Camptothecin. Cancer Res..

[17] Sordet O., Khan Q.A., Plo I., Pourquier P., Urasaki Y., Yoshida A., Antony S., Kohlhagen G., Solary E., Saparbaev M. (2004). Apoptotic Topoisomerase I-DNA Complexes Induced by Staurosporine-Mediated Oxygen Radicals. J. Biol. Chem..

[18] Pourquier P., Ueng L.M., Fertala J., Wang D., Park H.J., Essigmann J.M., Bjornsti M.A., Pommier Y. (1999). Induction of Reversible Complexes between Eukaryotic DNA Topoisomerase I and DNA-Containing Oxidative Base Damages. 7, 8-Dihydro-8-Oxoguanine and 5-Hydroxycytosine. J. Biol. Chem..

[19] Pourquier P., Bjornsti M.A., Pommier Y. (1998). Induction of Topoisomerase I Cleavage Complexes by the Vinyl Chloride Adduct 1, N6-Ethenoadenine. J. Biol. Chem..

[20] Ashour M.E., Atteya R., El-Khamisy S.F. (2015). Topoisomerase-Mediated Chromosomal Break Repair: An Emerging Player in Many Games. Nat. Rev. Cancer.

[21] Pommier Y., Barcelo J.M., Rao V.A., Sordet O., Jobson A.G., Thibaut L., Miao Z.H., Seiler J.A., Zhang H., Marchand C. (2006). Repair of Topoisomerase I-Mediated DNA Damage. Prog. Nucleic Acid Res. Mol. Biol..

[22] Pommier Y., Huang S.Y., Gao R., Das B.B., Murai J., Marchand C. (2014). Tyrosyl-DNA-Phosphodiesterases (TDP1 and TDP2). DNA Repair (Amst.).

[23] Kawale A.S., Povirk L.F. (2018). Tyrosyl-DNA Phosphodiesterases: Rescuing the Genome from the Risks of Relaxation. Nucleic Acids Res..

[24] Interthal H., Pouliot J.J., Champoux J.J. (2001). The Tyrosyl-DNA Phosphodiesterase Tdp1 is a Member of the Phospholipase D Superfamily. Proc. Natl. Acad. Sci. USA.

[25] El-Khamisy S.F., Saifi G.M., Weinfeld M., Johansson F., Helleday T., Lupski J.R., Caldecott K.W. (2005). Defective DNA Single-Strand Break Repair in Spinocerebellar Ataxia with Axonal Neuropathy-1. Nature.

[26] Takashima H., Boerkoel C.F., John J., Saifi G.M., Salih M.A., Armstrong D., Mao Y., Quiocho F.A., Roa B.B., Nakagawa M. (2002). Mutation of TDP1, Encoding a Topoisomerase I-Dependent DNA Damage Repair Enzyme, in Spinocerebellar Ataxia with Axonal Neuropathy. Nat. Genet..

[27] Interthal H., Chen H.J., Kehl-Fie T.E., Zotzmann J., Leppard J.B., Champoux J.J. (2005). SCAN1 Mutant Tdp1 Accumulates the Enzyme--DNA Intermediate and Causes Camptothecin Hypersensitivity. EMBO J..

[28] Meisenberg C., Gilbert D.C., Chalmers A., Haley V., Gollins S., Ward S.E., El-Khamisy S.F. (2015). Clinical and Cellular Roles for TDP1 and TOP1 in Modulating Colorectal Cancer Response to Irinotecan. Mol. Cancer Ther..

[29] Alagoz M., Wells O.S., El-Khamisy S.F. (2014). TDP1 Deficiency Sensitizes Human Cells to Base Damage via Distinct Topoisomerase I and PARP Mechanisms with Potential Applications for Cancer Therapy. Nucleic Acids Res..

[30] Roy A., Tesauro C., Frohlich R., Hede M.S., Nielsen M.J., Kjeldsen E., Bonven B., Stougaard M., Gromova I., Knudsen B.R. (2014). Decreased Camptothecin Sensitivity of the Stem-Cell-Like Fraction of Caco2 Cells Correlates with an Altered Phosphorylation Pattern of Topoisomerase I. PLoS ONE.

[31] Bandyopadhyay K., Li P., Gjerset R.A. (2012). CK2-Mediated Hyperphosphorylation of Topoisomerase I Targets Serine 506, Enhances Topoisomerase I-DNA Binding, and Increases Cellular Camptothecin Sensitivity. PLoS ONE.

[32] Murai J., Huang S.Y., Das B.B., Dexheimer T.S., Takeda S., Pommier Y. (2012). Tyrosyl-DNA Phosphodiesterase 1 (TDP1) Repairs DNA Damage Induced by Topoisomerases I and II and Base Alkylation in Vertebrate Cells. J. Biol. Chem..

[33] Jensen P.W., Falconi M., Kristoffersen E.L., Simonsen A.T., Cifuentes J.B., Marcussen L.B., Frohlich R., Vagner J., Harmsen C., Juul S. (2013). Real-Time Detection of TDP1 Activity using a Fluorophore-Quencher Coupled DNA-Biosensor. Biosens. Bioelectron..

[34] Fam H.K., Walton C., Mitra S.A., Chowdhury M., Osborne N., Choi K., Sun G., Wong P.C., O’Sullivan M.J., Turashvili G. (2013). TDP1 and PARP1 Deficiency are Cytotoxic to Rhabdomyosarcoma Cells. Mol. Cancer Res..

[35] Senter P.D., Beam K.S., Mixan B., Wahl A.F. (2001). Identification and Activities of Human Carboxylesterases for the Activation of CPT-11, a Clinically Approved Anticancer Drug. Bioconjug. Chem..

[36] Thomas A., Pommier Y. (2019). Targeting Topoisomerase I in the Era of Precision Medicine. Clin. Cancer Res..

[37] Gomez-Manzano C., Alonso M.M., Yung W.K., McCormick F., Curiel D.T., Lang F.F., Jiang H., Bekele B.N., Zhou X., Alemany R. (2006). Delta-24 Increases the Expression and Activity of Topoisomerase I and Enhances the Antiglioma Effect of Irinotecan. Clin. Cancer Res..

[38] Pavillard V., Charasson V., Laroche-Clary A., Soubeyran I., Robert J. (2004). Cellular Parameters Predictive of the Clinical Response of Colorectal Cancers to Irinotecan. A Preliminary Study. Anticancer Res..

[39] Meisenberg C., Ward S.E., Schmid P., El-Khamisy S.F. (2014). TDP1/TOP1 Ratio as a Promising Indicator for the Response of Small Cell Lung Cancer to Topotecan. J. Cancer Sci. Ther..

[40] Yu D., Khan E., Khaleque M.A., Lee J., Laco G., Kohlhagen G., Kharbanda S., Cheng Y.C., Pommier Y., Bharti A. (2004). Phosphorylation of DNA Topoisomerase I by the C-Abl Tyrosine Kinase Confers Camptothecin Sensitivity. J. Biol. Chem..

[41] Noach N., Segev Y., Levi I., Segal S., Priel E. (2007). Modification of Topoisomerase I Activity by Glucose and by O-GlcNAcylation of the Enzyme Protein. Glycobiology.

[42] Yu H., Park J., Lee J., Choi K., Choi C. (2012). Constitutive Expression of MAP Kinase Phosphatase-1 Confers Multi-Drug Resistance in Human Glioblastoma Cells. Cancer Res. Treat..

[43] Jandu H., Aluzaite K., Fogh L., Thrane S.W., Noer J.B., Proszek J., Do K.N., Hansen S.N., Damsgaard B., Nielsen S.L. (2016). Molecular Characterization of Irinotecan (SN-38) Resistant Human Breast Cancer Cell Lines. BMC Cancer.

[44] Rajapakse V.N., Luna A., Yamade M., Loman L., Varma S., Sunshine M., Iorio F., Sousa F.G., Elloumi F., Aladjem M.I. (2018). CellMinerCDB for Integrative Cross-Database Genomics and Pharmacogenomics Analyses of Cancer Cell Lines. iScience.

[45] Reinhold W.C., Sunshine M., Varma S., Doroshow J.H., Pommier Y. (2015). Using CellMiner 1.6 for Systems Pharmacology and Genomic Analysis of the NCI-60. Clin. Cancer Res..

[46] Comeaux E.Q., van Waardenburg R.C. (2014). Tyrosyl-DNA Phosphodiesterase I Resolves both Naturally and Chemically Induced DNA Adducts and its Potential as a Therapeutic Target. Drug Metab. Rev..

[47] Interthal H., Chen H.J., Champoux J.J. (2005). Human Tdp1 Cleaves a Broad Spectrum of Substrates, Including Phosphoamide Linkages. J. Biol. Chem..

[48] Zakharenko A.L., Luzina O.A., Sokolov D.N., Kaledin V.I., Nikolin V.P., Popova N.A., Patel J., Zakharova O.D., Chepanova A.A., Zafar A. (2019). Novel Tyrosyl-DNA Phosphodiesterase 1 Inhibitors Enhance the Therapeutic Impact of Topotesmall Es, Cyrillican on in Vivo Tumor Models. Eur. J. Med. Chem..

[49] Komarova A.O., Drenichev M.S., Dyrkheeva N.S., Kulikova I.V., Oslovsky V.E., Zakharova O.D., Zakharenko A.L., Mikhailov S.N., Lavrik O.I. (2018). Novel Group of Tyrosyl-DNA-Phosphodiesterase 1 Inhibitors Based on Disaccharide Nucleosides as Drug Prototypes for Anti-Cancer Therapy. J. Enzym. Inhib. Med. Chem..

[50] Lountos G.T., Zhao X.Z., Kiselev E., Tropea J.E., Needle D., Pommier Y., Burke T.R., Waugh D.S. (2019). Identification of a Ligand Binding Hot Spot and Structural Motifs Replicating Aspects of Tyrosyl-DNA Phosphodiesterase I (TDP1) Phosphoryl Recognition by Crystallographic Fragment Cocktail Screening. Nucleic Acids Res..

[51] Zhang X.R., Wang H.W., Tang W.L., Zhang Y., Yang H., Hu D.X., Ravji A., Marchand C., Kiselev E., Ofori-Atta K. (2018). Discovery, Synthesis, and Evaluation of Oxynitidine Derivatives as Dual Inhibitors of DNA Topoisomerase IB (TOP1) and Tyrosyl-DNA Phosphodiesterase 1 (TDP1), and Potential Antitumor Agents. J. Med. Chem..

[52] Desai S.D., Li T.K., Rodriguez-Bauman A., Rubin E.H., Liu L.F. (2001). Ubiquitin/26S Proteasome-Mediated Degradation of Topoisomerase I as a Resistance Mechanism to Camptothecin in Tumor Cells. Cancer Res..

[53] Beidler D.R., Cheng Y.C. (1995). Camptothecin Induction of a Time- and Concentration-Dependent Decrease of Topoisomerase I and its Implication in Camptothecin Activity. Mol. Pharmacol..

[54] Hudson J.J., Chiang S.C., Wells O.S., Rookyard C., El-Khamisy S.F. (2012). SUMO Modification of the Neuroprotective Protein TDP1 Facilitates Chromosomal Single-Strand Break Repair. Nat. Commun..

[55] Chiang S.C., Carroll J., El-Khamisy S.F. (2010). TDP1 Serine 81 Promotes Interaction with DNA Ligase IIIalpha and Facilitates Cell Survival Following DNA Damage. Cell Cycle.

[56] Das B.B., Antony S., Gupta S., Dexheimer T.S., Redon C.E., Garfield S., Shiloh Y., Pommier Y. (2009). Optimal Function of the DNA Repair Enzyme TDP1 Requires its Phosphorylation by ATM and/Or DNA-PK. EMBO J..

[57] Das B.B., Huang S.Y., Murai J., Rehman I., Ame J.C., Sengupta S., Das S.K., Majumdar P., Zhang H., Biard D. (2014). PARP1-TDP1 Coupling for the Repair of Topoisomerase I-Induced DNA Damage. Nucleic Acids Res..

[58] Berti M., Ray Chaudhuri A., Thangavel S., Gomathinayagam S., Kenig S., Vujanovic M., Odreman F., Glatter T., Graziano S., Mendoza-Maldonado R. (2013). Human RECQ1 Promotes Restart of Replication Forks Reversed by DNA Topoisomerase I Inhibition. Nat. Struct. Mol. Biol..

[59] Regairaz M., Zhang Y.W., Fu H., Agama K.K., Tata N., Agrawal S., Aladjem M.I., Pommier Y. (2011). Mus81-Mediated DNA Cleavage Resolves Replication Forks Stalled by Topoisomerase I-DNA Complexes. J. Cell Biol..

[60] Zhang Y.W., Regairaz M., Seiler J.A., Agama K.K., Doroshow J.H., Pommier Y. (2011). Poly(ADP-Ribose) Polymerase and XPF-ERCC1 Participate in Distinct Pathways for the Repair of Topoisomerase I-Induced DNA Damage in Mammalian Cells. Nucleic Acids Res..

[61] Das S.K., Rehman I., Ghosh A., Sengupta S., Majumdar P., Jana B., Das B.B. (2016). Poly(ADP-Ribose) Polymers Regulate DNA Topoisomerase I (Top1) Nuclear Dynamics and Camptothecin Sensitivity in Living Cells. Nucleic Acids Res..

[62] Meisenberg C., Ashour M.E., El-Shafie L., Liao C., Hodgson A., Pilborough A., Khurram S.A., Downs J.A., Ward S.E., El-Khamisy S.F. (2017). Epigenetic Changes in Histone Acetylation Underpin Resistance to the Topoisomerase I Inhibitor Irinotecan. Nucleic Acids Res..

[63] Antony S., Marchand C., Stephen A.G., Thibaut L., Agama K.K., Fisher R.J., Pommier Y. (2007). Novel High-Throughput Electrochemiluminescent Assay for Identification of Human Tyrosyl-DNA Phosphodiesterase (Tdp1) Inhibitors and Characterization of Furamidine (NSC 305831) as an Inhibitor of Tdp1. Nucleic Acids Res..

[64] Simplicio A.L., Coroadinha A.S., Gilmer J.F., Lamego J. (2013). A Methodology for Detection and Quantification of Esterase Activity. Methods Mol. Biol..

