# Supplementary material for: Tyrosyl-DNA Phosphodiesterase 1 and Topoisomerase I Activities as Predictive Indicators for Glioblastoma Susceptibility to Genotoxic Agents
Source: Cancers (Basel). 2019 Sep 23;11(10):1416. doi: 10.3390/cancers11101416 (PMC6827102; doi:10.3390/cancers11101416)
Supplement: Supplementary file 1 [file cancers-11-01416-s001.zip › Cancers Supplementary Figures_Revised.docx]

Supplementary Materials: Tyrosyl-DNA Phosphodiesterase 1 and Topoisomerase I Activities as Predictive Indicators for Glioblastoma Susceptibility to Genotoxic Agents

Wenjie Wang ^1,2^, Monica Rodriguez-Silva ^3^ Arlet M. Acanda de la Rocha ^3^, Aizik L. Wolf ^4^, Yanhao Lai ^1,2^, Yuan Liu ^1,2^, William C. Reinhold ^5^, Yves Pommier ^5^, Jeremy W. Chambers ^1,3,^* and Yuk-Ching Tse-Dinh ^1,2,^*


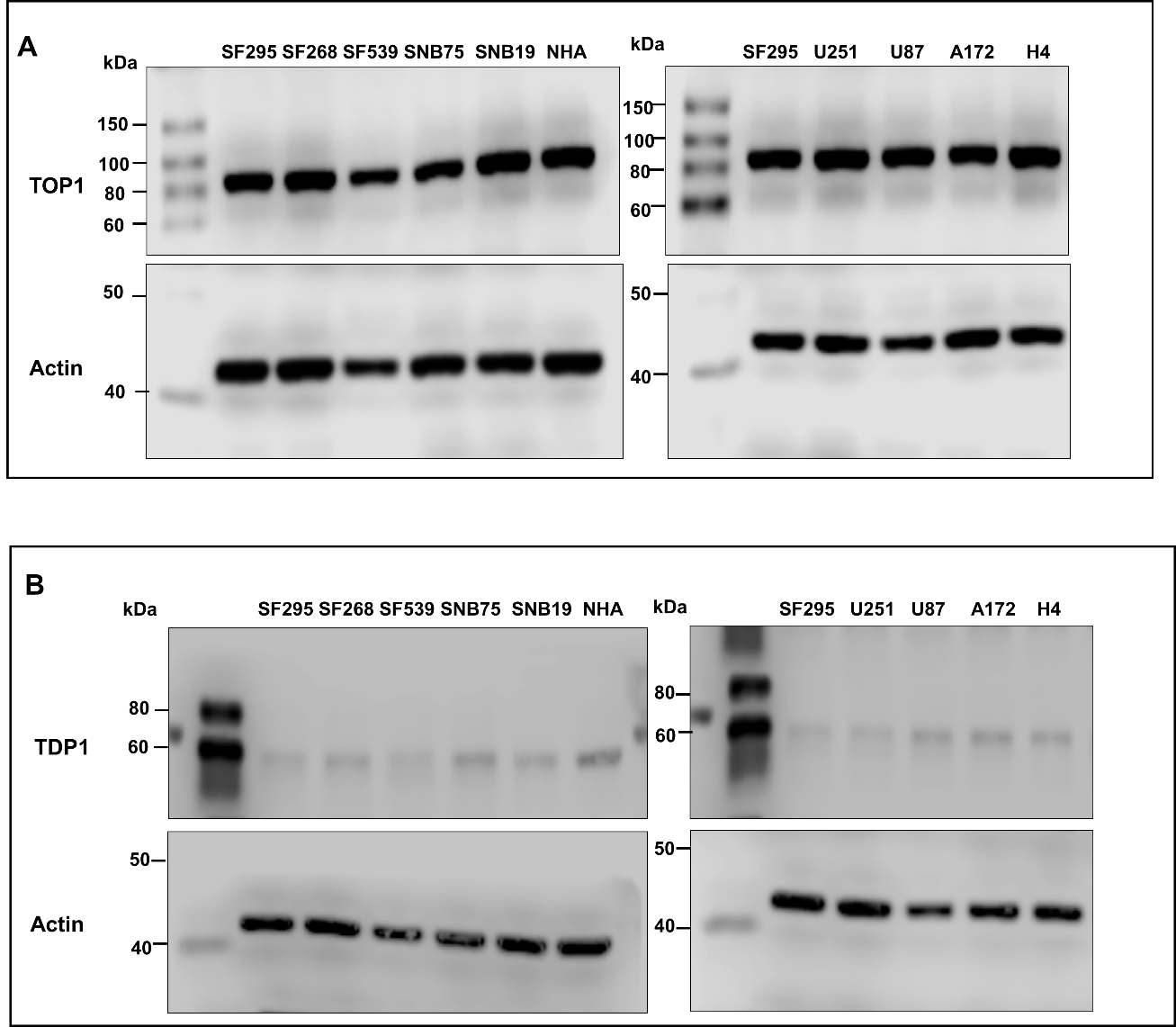


**Figure S1.** Blots from Figure 2A and Figure 2B.


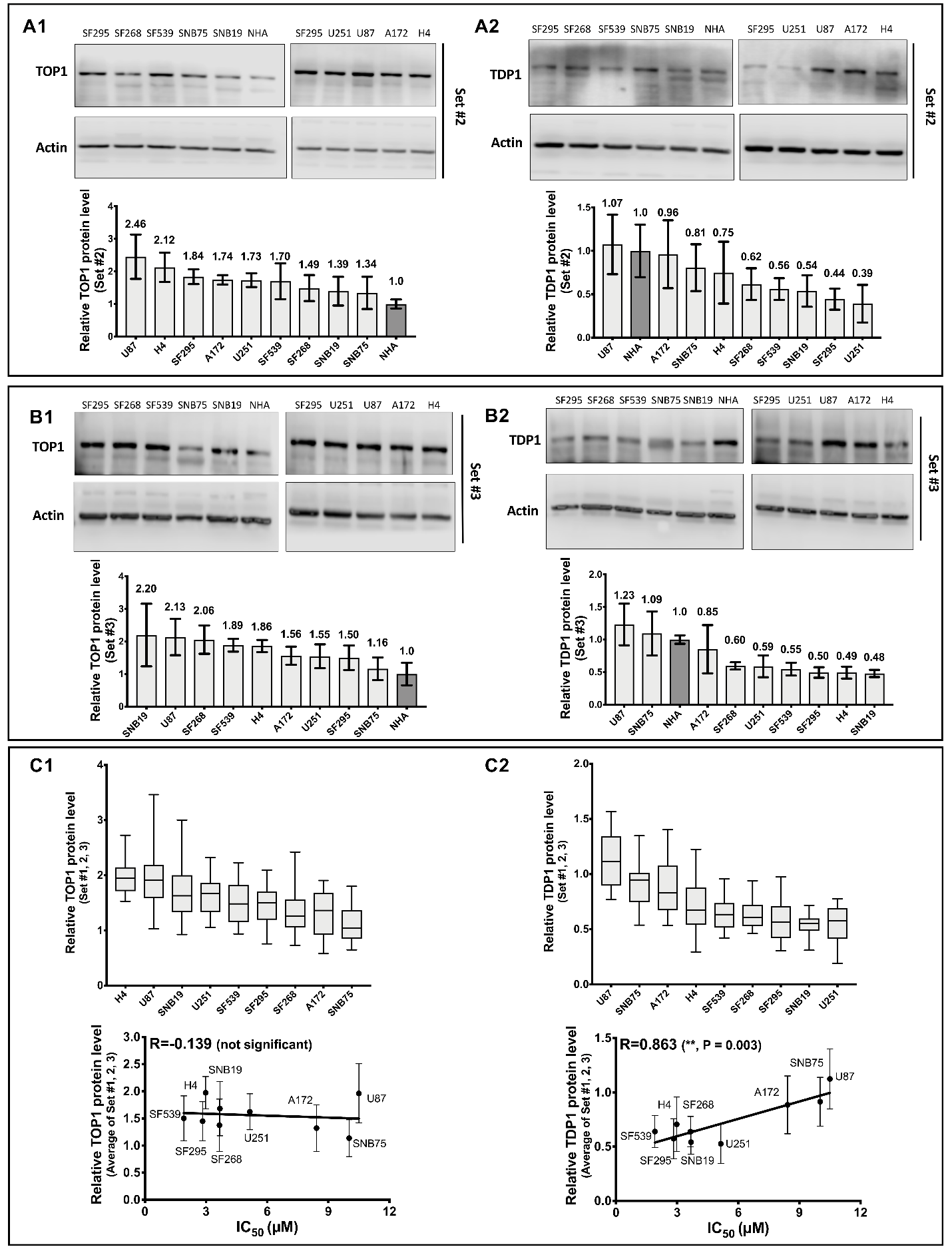


**Figure S2.** TOP1 and TDP1 protein levels measurements in different sets of GBM cell lines to assess potential correlation with irinotecan IC_50_. Relative levels of TOP1 protein expression in (A1) set #2 and (B1) set #3 GBM cell lines were compared against NHA by Western blotting. Relative levels of TDP1 protein expression in (A2) set #2 and (B2) set #3 were also compared by Western blotting against NHA. Signal from actin was used to control the loading of WCE (10 µg total protein). The bar graphs show the average and standard deviation for at least 3 measurements in each set of experiment. The box plots in (C1, C2) show the median of data from all three sets of experiments with minimum and maximum values at the bottom and top tail-ends. Pearson correlations between irinotecan IC_50_ values and relative TOP1 protein level (C1) or relative TDP1 protein levels (C2) are shown for the average of set #1, #2, #3 GBM cell lines.


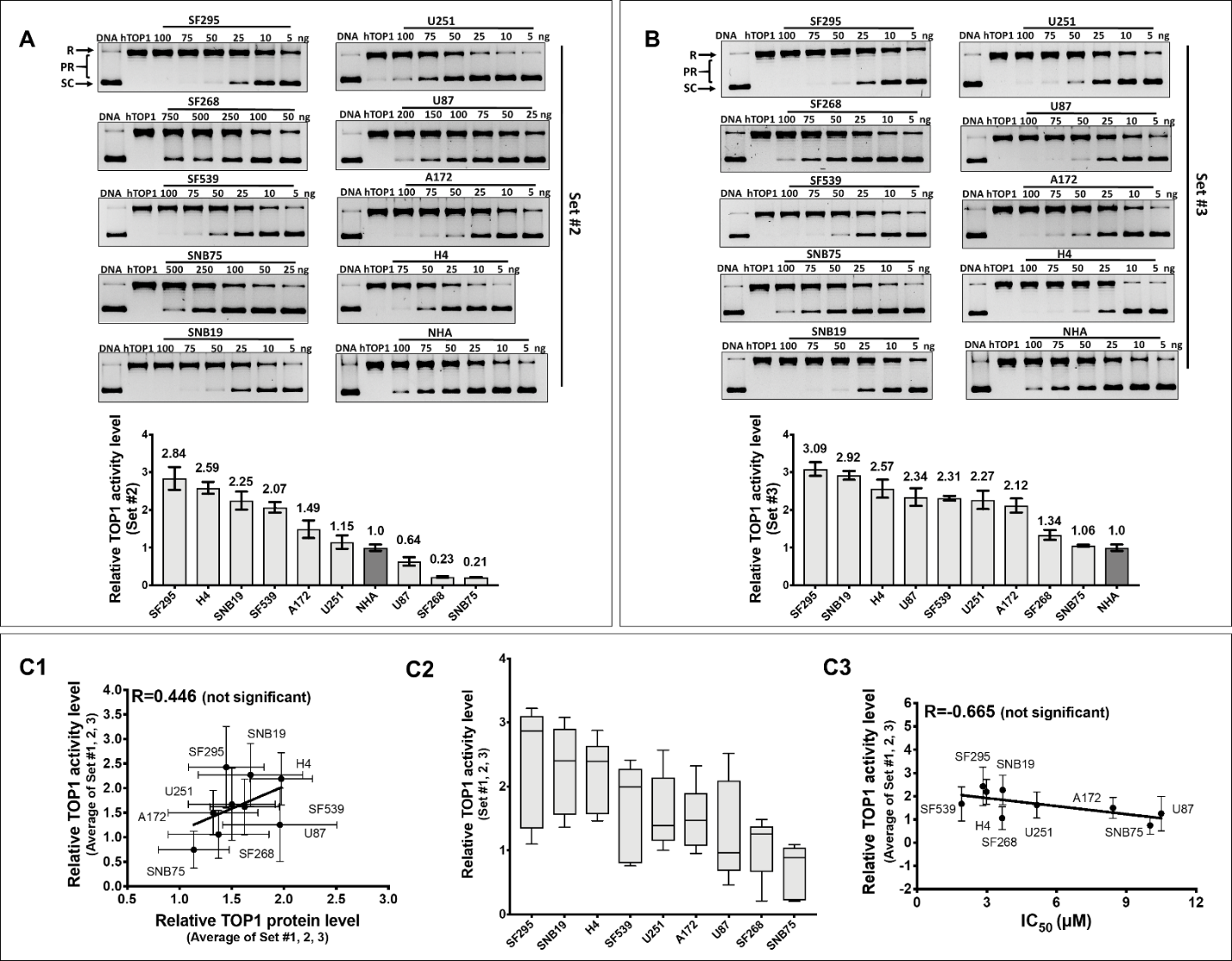


**Figure S3.** Topoisomerase I (TOP1) activity in replicated sets of GBM cell line experiments does not correlate with TOP1 protein levels or irinotecan IC_50_. Relative levels of TOP1 activity in (A) set #2 and (B) set #3 GBM cell lines were compared against NHA by plasmid DNA relaxation assay. R: relaxed DNA; PR: partially relaxed DNA; SC: supercoiled DNA. The bar graphs show the average and standard deviation for at least 3 measurements in each set of experiment. (C1) Pearson correlation between relative TOP1 protein levels and relative TOP1 activity levels are shown for the average of set #1, #2, #3 GBM cell lines. The box plot in (C2) shows the median of relative TOP1 activity from all three sets of experiments with minimum and maximum values at the bottom and top tail-ends. (C3) Pearson correlation between irinotecan IC_50_ values and relative TOP1 activity levels are shown for the average of replicates set #1, #2, #3 GBM cell lines.


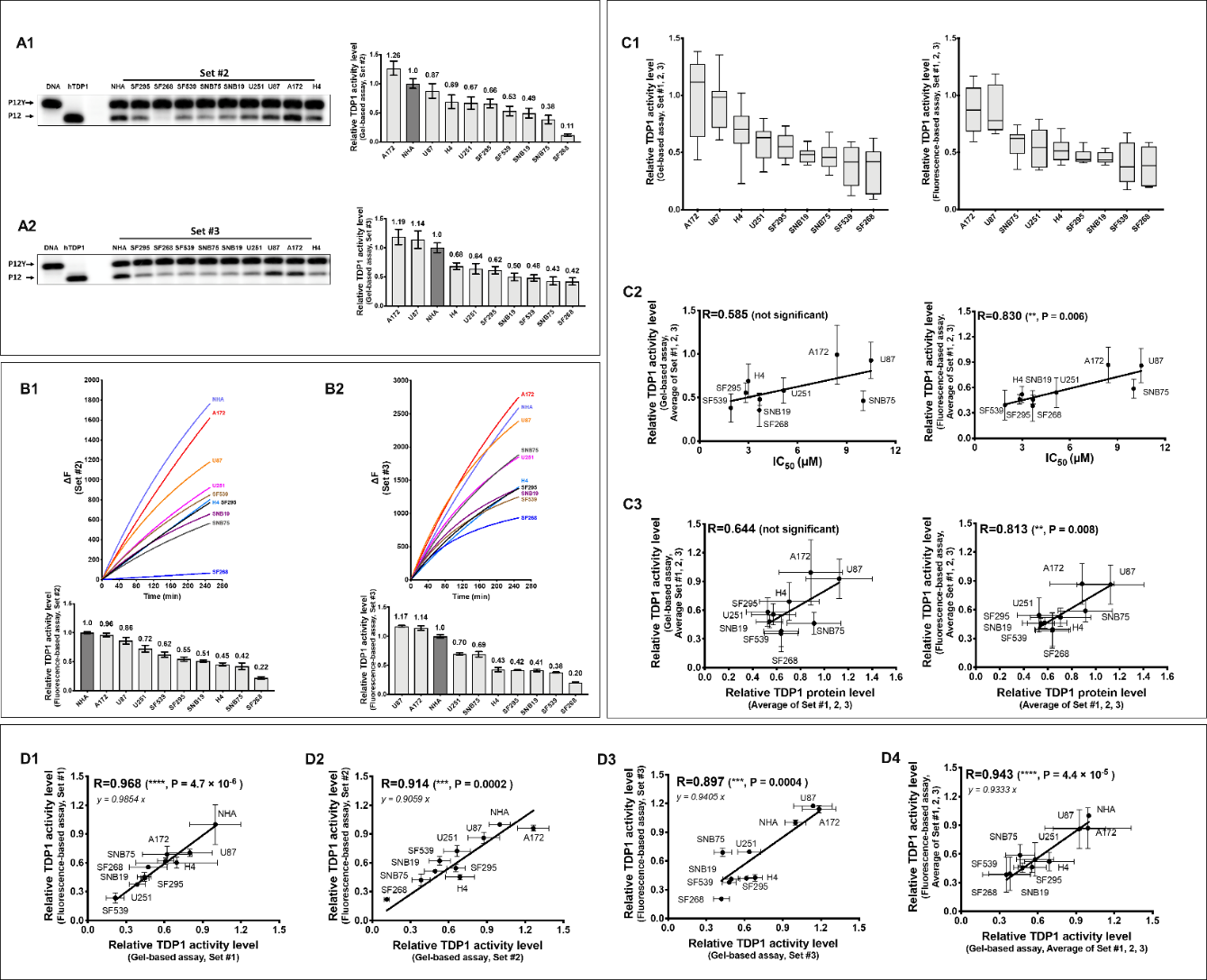


**Figure S4. TDP1 activity levels show slight correlation with irinotecan IC_50_.** Relative levels of TDP1 activity in (A1, B1) replicates set #2 and (A2, B2) set #3 were compared by gel-based assay and fluorescence-based assay against NHA. P12Y: radiolabeled DNA substrate; P12: product formed by TDP1. The bar graphs show the average and standard deviation for at least 3 measurements in each set of experiment. The box plots in (C1) show the median of relative TDP1 activity from all three sets of experiments with minimum and maximum values at the bottom and top tail-ends. Pearson correlations between the average of relative TDP1 activity from set #1, #2, #3 and irinotecan IC_50_ values are shown in (C2). Pearson correlation between relative TDP1 protein levels and TDP1 activity levels are shown in (C3) for the average from set #1, #2, #3 experiments. Pearson correlation between TDP1 activity levels measured by gel-based assay and fluorescence-based assay are shown for (D1) set #1, (D2) set #2, (D3) set #3, and (D4) average of all three sets of replicated experiments on the GBM cell lines.


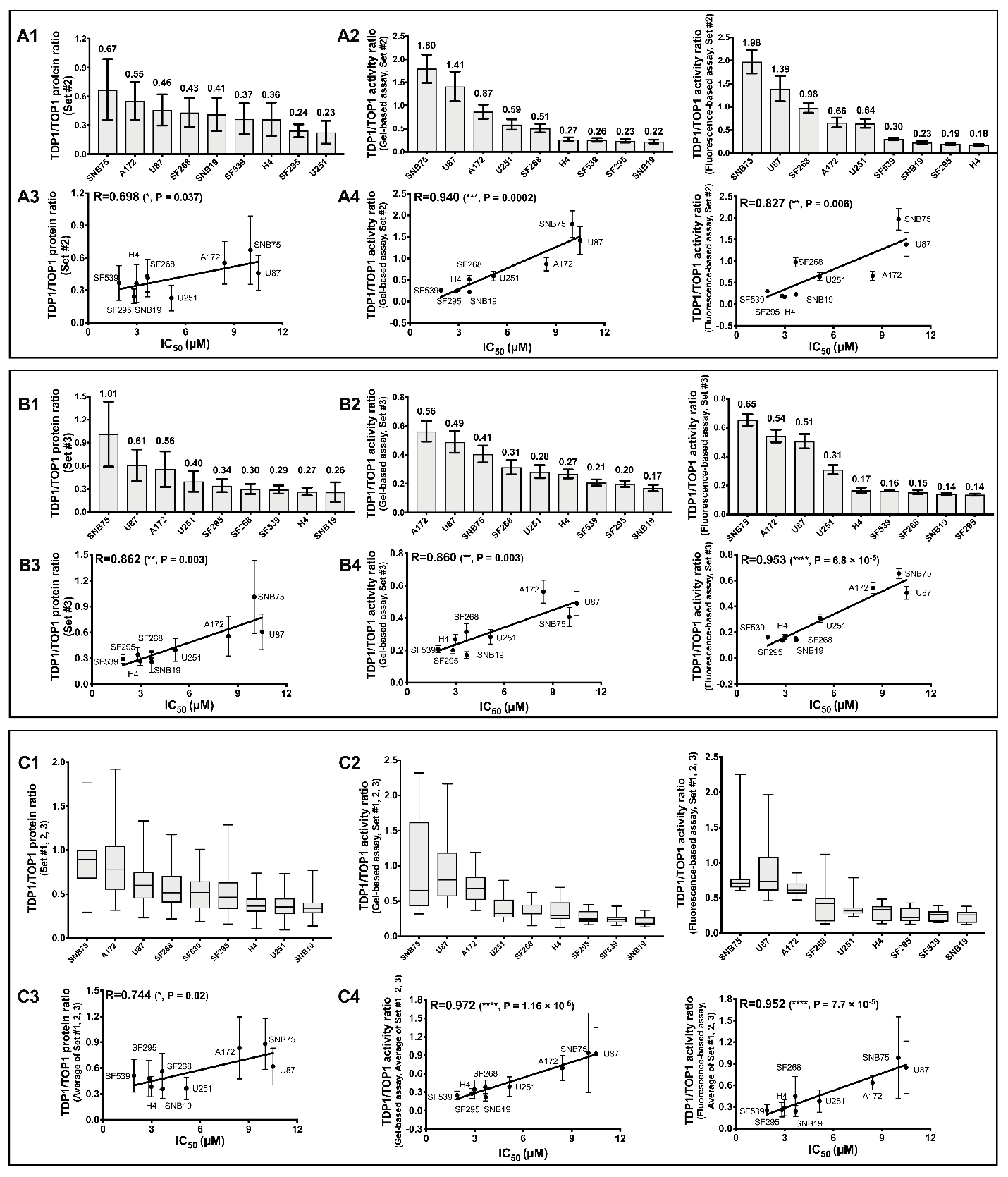


**Figure S5. TDP1/TOP1 activity ratio is a stronger predictor than TDP1/TOP1 protein ratio for irinotecan IC_50_.** Relative TDP1/TOP1 protein ratios are shown for (A1) set #2 and (B1) set #3 experiments. Relative TDP1/TOP1 activity ratios from the gel-based and fluorescence-based TDP1 activity assay are shown for (A2) set #2 and (B2) set #3. The box plots show the median of (C1) TDP1/TOP1 protein or (C2) activity ratios from all three sets of experiments with minimum and maximum values at the bottom and top tail-ends. Pearson correlations between the TDP1/TOP1 protein ratios and irinotecan IC_50_s are shown for (A3) set #2, (B3) set #3 and (C3) the average from set #1, 2, 3. Pearson correlations between the TDP1/TOP1 activity ratios and irinotecan IC_50_s are shown for (A4) set #2, (B4) set #3 and (C4) the average from replicated experiments set #1, 2, 3. .


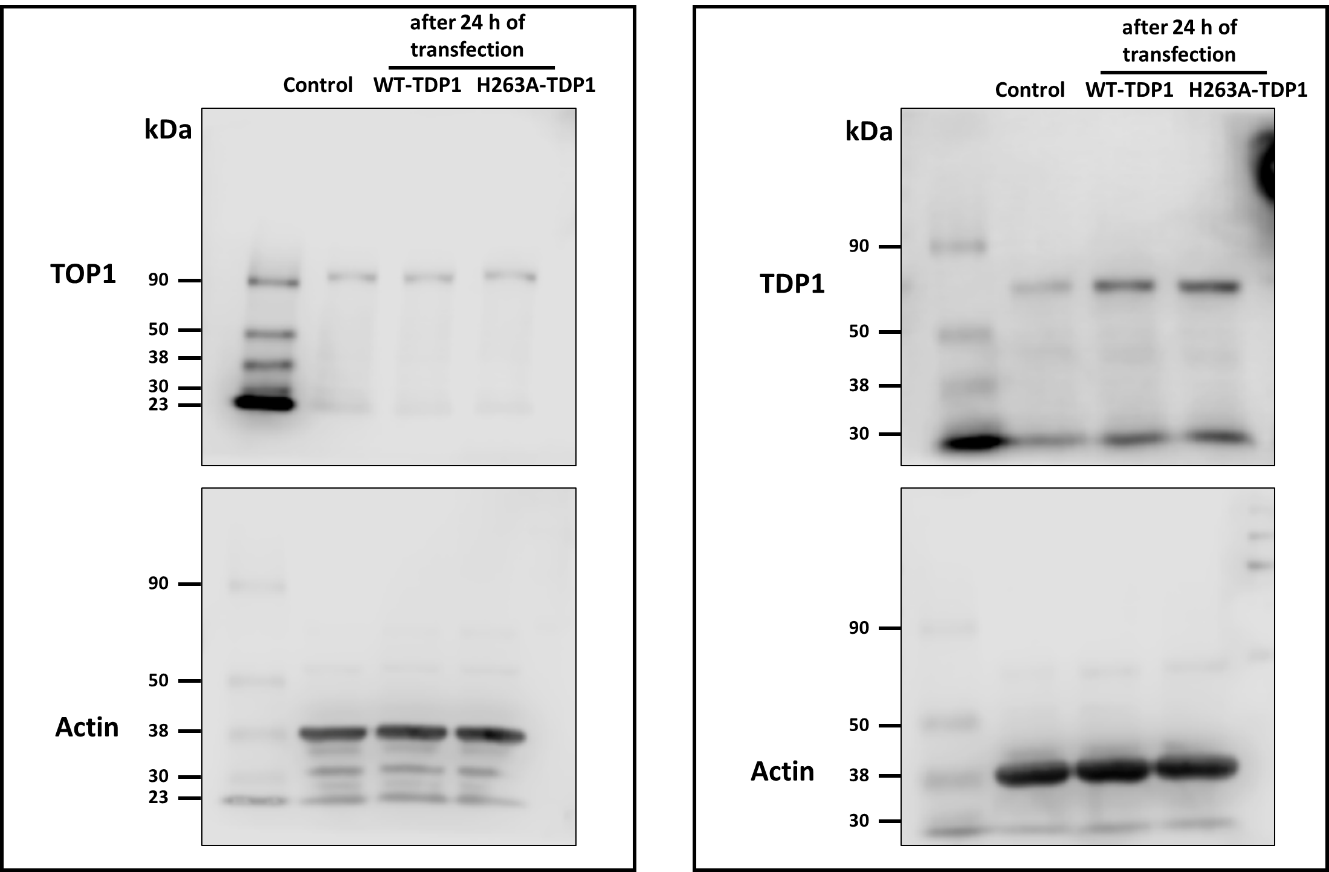


**Figure S6.** Blots from Figure 6A.


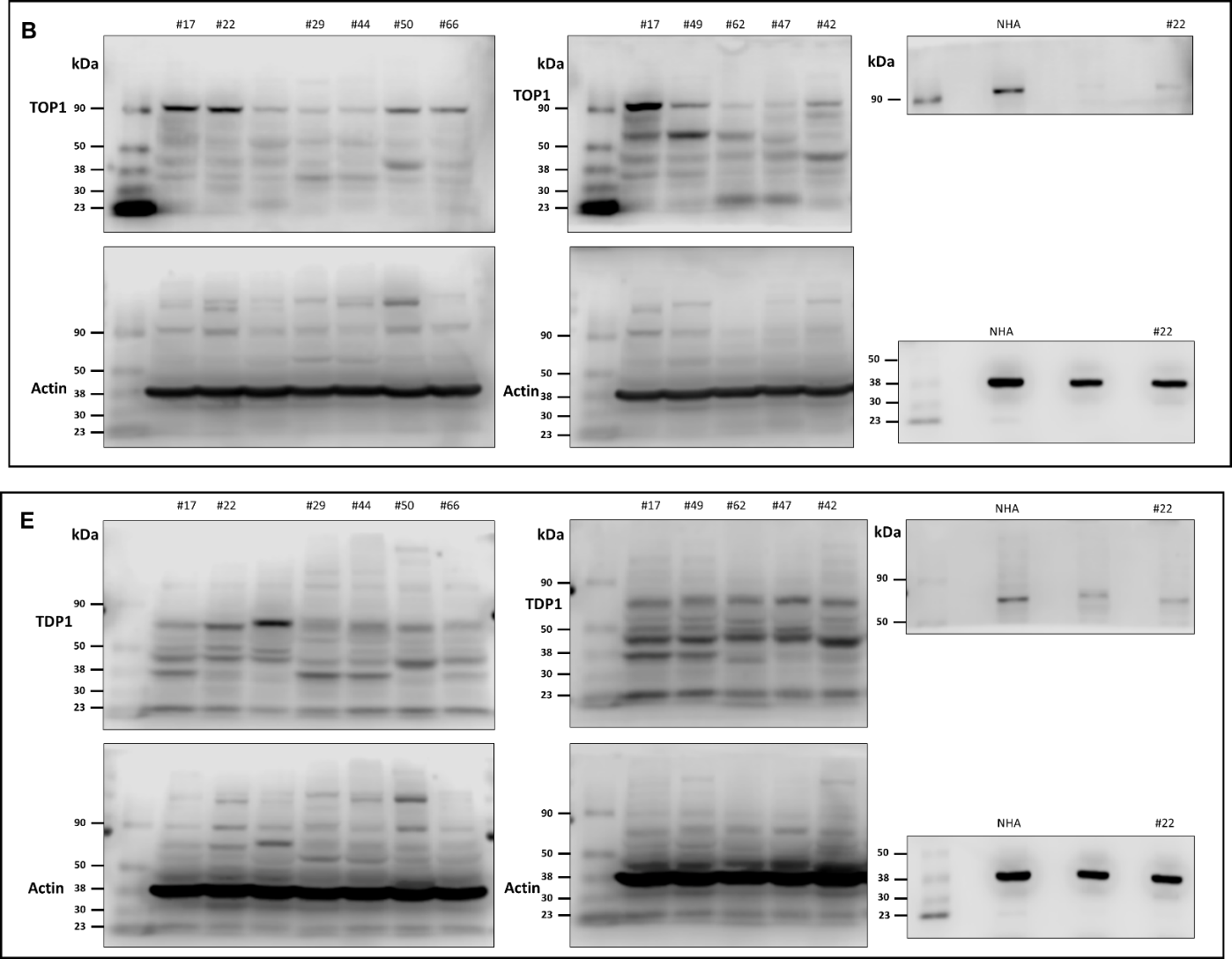


**Figure S7.** Blots from Figure 8B and 8E.
